# A Brief Overview of Ethanol Tolerance and Its Potential Association with Circadian Rhythm in *Drosophila*

**DOI:** 10.3390/ijms252312605

**Published:** 2024-11-24

**Authors:** Sophie K. Peterson, S. Tariq Ahmad

**Affiliations:** Department of Biology, Colby College, Waterville, ME 04901, USA; skpete25@colby.edu

**Keywords:** ethanol-induced behaviors, tolerance, circadian rhythm, genetics, *Drosophila*

## Abstract

Alcohol consumption and addiction remain global health concerns, with significant loss of productivity, morbidity, and mortality. *Drosophila melanogaster*, a widely used model organism, offers valuable insights into the genetic and neuronal mechanisms underlying ethanol-induced behaviors (EIBs) such as sedation, recovery, and tolerance. This narrative review focuses on studies in the *Drosophila* model system suggesting an association between circadian rhythm genes as modulators of ethanol tolerance. Mutations in these genes disrupt both the circadian cycle and tolerance, underscoring the interplay between circadian rhythm and ethanol processing although the exact mechanisms remain largely unknown. Additionally, genes involved in stress response, gene expression regulation, neurotransmission, and synaptic activity were implicated in ethanol tolerance modulation. At the neuronal level, recent studies have highlighted the involvement of corazonin (CRZ) and neuropeptide F (NPF) neurons in modulating EIBs. Understanding the temporal dynamics of tolerance development is crucial for describing the molecular basis of ethanol tolerance. Ultimately, insights gained from *Drosophila* studies hold promise for elucidating the neurobiological underpinnings of alcohol use disorders and addiction, contributing to more effective interventions and treatments.

## 1. Introduction

Alcohol is one of the most commonly used mind-altering substances in the world. Alcohol use has a significant negative impact on the health and well-being of humans. In 2019, ethanol consumption was responsible for 2.37 million deaths worldwide [1]. *Drosophila melanogaster*, or the common fruit fly, rose in popularity as a model for human ethanol consumption and processing in the last quarter of the 20th century with the demonstrations that the physiological and behavioral effects of ethanol in *Drosophila* are excellent parallels to those of humans [2]. Studies investigating the genes influencing ethanol behaviors in *Drosophila* began appearing in publications at the end of the 20th century with publications in the field doubling by the end of the first decade of the 21st century. Tolerance to ethanol is one of the most commonly studied ethanol-related behaviors. However, tolerance is defined differently based on the field/context of the study. In the fields of evolutionary biology and ecology, tolerance generally means innate resistance to alcohol intoxication in naïve animals. In addiction biology, tolerance is defined as a change in behavior upon repeated exposure to alcohol, which is the primary focus of this review.

*Drosophila* inhabit and thrive in ethanol-rich environments, such as those found around overripe fruits that contain up to 5% ethanol produced by the fermentation of sugars by yeast [3,4]. Studies have shown that yeast is the preferred food source for *Drosophila* larvae, and thus ethanol is found in high concentrations in the most nutrient-rich locations for *Drosophila* [5,6]. *Drosophila* are known to inhabit wineries, where the ethanol concentration can reach up to 11% [3]. The functions of alcohol dehydrogenase (ADH) and acetaldehyde dehydrogenase (ALDH) are essential in metabolizing ethanol and thus minimizing its toxic effects in both humans and fruit flies [7]. *Drosophila* ADH is a short-chain dehydrogenase compared to the more common medium-chain isoform, and it is hypothesized that this isoform of the enzyme evolved as a response to high ethanol concentrations in *Drosophila* habitats [8]. Beyond their natural connection to ethanol, *Drosophila* is an ideal animal model because of its small size, fecundity, and short 10-day lifecycle, which allows for assays to be conducted on large scales in short time spans [8]. *Drosophila* is a particularly apt model for studies that focus on the genetic basis of human behavior and disorders, as roughly 75% of human disease-causing genes have orthologs in *Drosophila* [9]. Ethanol interacts with multiple proteins and pathways, which makes a model organism whose entire genome can be analyzed easily and efficiently all the more important. The wide availability of mutants, gene libraries, tools for regulating gene expression (Gal4-UAS flies, RNA interference, CRISPR, etc.), and simple mutation induction/genetic screens allow for the creation of unique genotypes and the upregulation and downregulation of gene expression in *Drosophila*. Many genome-wide association studies (GWAS) have also been conducted in *Drosophila* to identify genes that affect EIBs [10,11,12].

## 2. Ethanol-Induced Behaviors in *Drosophila*

Upon ethanol exposure, *Drosophila*, similar to humans and mammals, demonstrate increased locomotion at low doses, and eventual sedation at higher doses of ethanol [13,14,15]. The most commonly studied ethanol-induced behaviors (EIBs) in *Drosophila* are sedation, recovery, and tolerance. Sedation is defined as the time at which *Drosophila* become unresponsive to mechanical stimulation, lose postural control, locomotion, and experience a loss of righting reflex (LORR) [16]. Recovery time reflects the time period from the end of exposure to the point at which flies have recovered from the sedative effects. In most studies, this time is defined as when the fly regains LORR and/or locomotion after sedation.

Tolerance is the development of an exposure-mediated endurance to the sedating effects of ethanol that extends the alert period and decreases recovery time. Pharmacokinetic (metabolic) tolerance identifies the change in the rate of drug metabolism due to prior exposures, but this is not as applicable to the *Drosophila* model as adult flies have not been shown to develop pharmacokinetic tolerance [17,18]. Pharmacodynamic or functional tolerance refers to the tolerance due to neurophysiological changes and can be divided into acute, rapid, and chronic categories. Acute tolerance develops during an initial exposure itself, rapid tolerance develops after a single, short-term ethanol exposure, and chronic tolerance develops after a prolonged exposure or multiple exposures [16,17]. *Drosophila* are a particularly suitable model for studying rapid tolerance, which can be observed (at varying strengths) from anywhere between 2–36 h after an initial sedation [18,19]. This narrative review is focused on rapid tolerance, which appears to be the most commonly studied.

Ethanol-induced behaviors in *Drosophila* are commonly studied using assays that involve respiration-mediated absorption of ethanol vapor, which does cause differential responses at different concentrations and exposure times but is not necessarily a direct parallel to voluntary consumption of ethanol [20]. While the vapor method may not be an ideal model, it does allow for forced consumption, which can make dosages simpler and more consistent. There are several popular assays used to quantify EIBs in *Drosophila*. The first automated technique used to measure the sedation of *Drosophila* utilized the inebriometer—a vertical glass column with a continuous flow of ethanol vapor [15,21,22]. The column contains a series of spiraling ledges which flies slide down as they become increasingly intoxicated. When flies fall through the bottom of the column, they are counted by a machine. This device measures the time at which flies become sufficiently uncoordinated and lose the ability to balance, climb, or stand [21,23].

Another commonly used assay involves tubes/vials with liquid ethanol on the bottom of cotton plugs/caps as the source of ethanol vapor. Vials are periodically tapped to observe and manually record the flies experiencing LORR or other related behaviors. These assays quantify a timeline of sedation, usually between 3–8 min, depending on several factors [24,25]. The most commonly used quantification of data is the time for half of the flies in a given trial to be sedated, or experience LORR (ST50). However, a drawback of such assays is the subjective scoring of the behaviors by the experimenter. Several assays are emerging that modify these manual assays with the use of a camera and software-mediated automated tracking of locomotion to provide more exact data and eliminate human error [26,27]. Both manual and automated tracking assays can be used to determine rapid tolerance to ethanol if a second exposure is performed some time later after the first exposure, usually 4–24 h. The increase in time to reach ST50 during this second sedation can be described as rapid tolerance [17]. Currently, the most common experimental paradigm to identify genes that alter ethanol-induced behaviors is to document the changes in temporal attributes of these behaviors in genetically modified flies (via mutant allele, RNAi-mediated knockdown, CRISPR, and/or ectopic overexpression). The change in timing of EIBs in flies with altered expression suggests that the gene product plays a role in modulating the behavior(s).

The *Drosophila* model is also used to observe voluntary and preferential consumption of ethanol. Although preference is an ethanol-related behavior with important implications across several fields of study, it is not an ethanol-induced behavior (does not necessarily result from prior ethanol exposure) and thus is beyond the scope of this review. Preference, sedation, and multiple forms of tolerance in the *Drosophila* model are often used to study the genetic basis of Alcohol Use Disorders (AUDs) and addiction in humans [28]. Several reviews in recent years have described differential gene expression due to ethanol exposure as it relates to AUDs [16,29,30]. While these ever-growing fields are important to our understanding of addiction development, this review focuses on genetic modifiers of rapid tolerance. Understanding these mechanisms is informative about the genetic basis of tolerance and other EIBs.

## 3. Neuronal Basis of Ethanol Tolerance

Because synaptic plasticity is involved in the formation of addictive behaviors, it is crucial to understand the cellular and molecular pathways that underlie synaptic plasticity if we are to truly understand ethanol tolerance (recently reviewed in [31]). Neuronal groups such as pars intercerebralis, the ventral subesophageal ganglion, and the dorsal giant neurons were shown to produce ethanol sensitivity, which relates to sedation [32,33]. Corazonin (CRZ) neurons were identified as modulators of EIBs [34,35,36]. CRZ-expressing neurons were shown to affect ethanol sensitivity, recovery, and preference. CRZ neurons also modulate other reward pathways such as ejaculation and copulation, suggesting that its regulation of EIBs may also be on a reward-dependent basis [34,37]. Neurons expressing Neuropeptide F (NPF, *Drosophila* ortholog of human NPY) were also shown to modify EIBs, with a specific effect on sedation modification, potentially via the protein kinase C pathway [38,39,40,41]. NPF is thought to be connected to CRZ in that activation of CRZ neurons causes an increase in NPF levels [42,43]. There have also been several studies on the neuronal basis of ethanol preference in *Drosophila*, which include dopaminergic neurons, octopaminergic neurons, mushroom body neurons, serotonergic neurons, etc. [44,45,46,47]. Although there is no direct investigation on tolerance modulation in any of these studies, these neuronal groups are promising candidate circuits and warrant further investigation.

**Figure 1:**
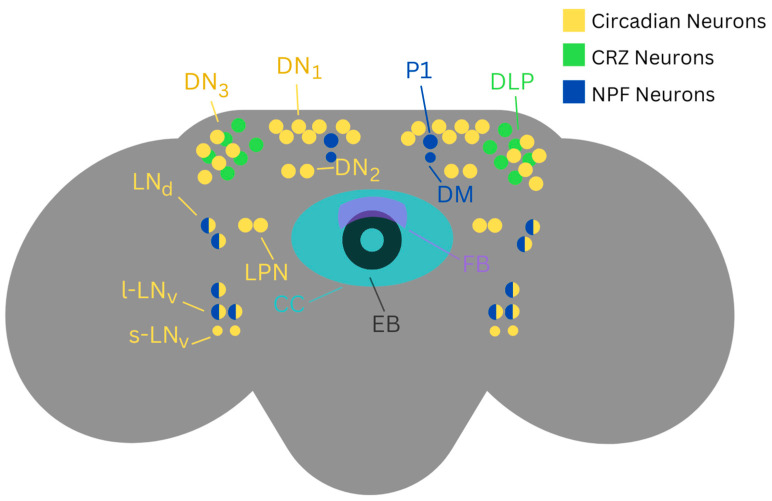
Diagram of the *Drosophila* adult brain depicting the approximate location of brain structures and neuronal clusters that are seemingly associated with the modulation of ethanol tolerance. These include Central Complex (CC; teal) [18,48,49,50], Ellipsoid Body (EB; black) [51], fan body [50], core clock neurons (yellow) [41,52,53], Corazonin-expressing neurons (CRZ; green) [34,35,36,37], and NPF-expressing neurons (NPF; blue) [38,39,40,41]. The primary circadian neuronal network is composed of extensively interconnected Lateral Neurons (s-LNv, l-LNv, LNd, and LPN) and Dorsal Neurons (DN1, DN2, DN3). Many of these neuronal clusters project fibers into the dorsal protocerebrum which includes neurosecretory centers in the pars intercerebralis (PI) and pars lateralis (PL) as well as into the accessory medulla (AME) [52,53]. CRZ expression is primarily confined to 6–8 neurons in the endocrine Dorso-Lateral Peptidergic (DLP) clusters [34,36]. NPF is primarily expressed in the large (P1) and small (DM) dorsal medial neurons, large dorsal lateral neurons (LNd), small fan-shaped body (FB; purple) [40], and lateral ventral neurons (LNv) [41]. Neuronal clusters are labeled on one side of the medial–lateral axis of symmetry.

There has been some success in identifying the neuronal basis of tolerance. Multiple studies have found a connection between the central complex and ethanol tolerance, and some have gone as far as to identify specific neuronal groups as tolerance modulators within the central complex [18,48,49,50]. Additionally, ellipsoid body neurons have been implicated in the creation of ethanol tolerance via a GPCR Kinase 2 pathway [51]. Although the central complex does not have much overlap with CRZ- and NPF-expressing neurons, there seems to be an overlap between CRZ- and NPF-expressing neurons and core clock neurons [52,53] (Figure 1).

## 4. Genetic Basis of Ethanol Tolerance

**Figure 2:**
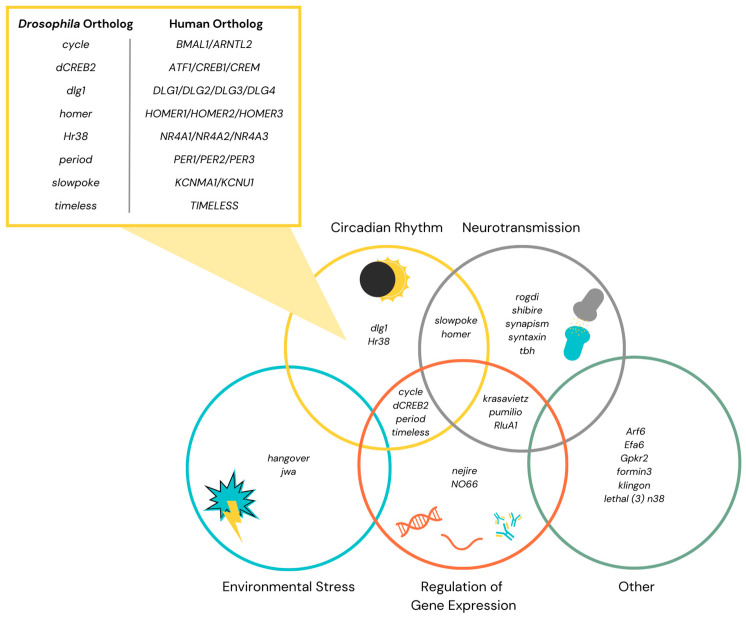
Venn diagram illustrating the genes currently associated with the modulation of ethanol tolerance and the functional categories to which they belong. Inset lists *Drosophila* core circadian and clock-controlled genes with circadian expression and their human orthologs. Notes: The placement of genes in the Venn diagram does not exhaustively represent their putative functions. Graphics represent these functional categories. See Park et al., 2017 [23] for a more comprehensive illustration of association of genes with ethanol tolerance, resistance, and preference.

Several physiological categories are apparent among the genes currently implicated in altering the tolerance phenotype. Many tolerance-altering genes play roles in circadian rhythm, synaptic plasticity or neurotransmission, environmental stress pathways, or regulation of gene expression, although not every gene falls into one of these categories (Figure 2). Many of these genes have several roles and most studies do not provide clear associations between tolerance modulation and their canonical function. Also, a recent study has identified an inverse correlation between initial sedation time and ethanol tolerance, suggesting that the tolerance phenotype exhibited by mutants with increased initial sensitivity or resistance to ethanol might be due to variation in this initial sedation rather than a true difference in tolerance development [54]. It is important to take this finding into consideration when analyzing genes for effects on tolerance. Nevertheless, the wide array of gene types involved in tolerance suggests a variety of mechanisms of interaction between these genes and ethanol and there is still a lot to be understood about the many genetic connections to tolerance. It is valuable to identify the functional categories of genes connected with tolerance. In this section, we have briefly mentioned some studies as examples of various functional categories of genes having an effect on tolerance. In the following section, we have highlighted the studies carried out in *Drosophila* to make a case for the association between circadian genes and tolerance (Figure 2).

Genes that respond to environmental stress regularly appear in genetic screenings for EIB modulators. Perhaps one of the most well-known modulators of genes in ethanol behavior, *hangover*, which encodes a zinc-finger protein often connected to nucleic acid binding, belongs in this stress response category [55,56]. Flies with the mutant *hangover* allele have reduced ethanol tolerance and a defective response to some environmental stressors such as heat and free radicals [56]. *jwa*, which encodes a microtubule-binding protein that regulates the intracellular transport of amino acids, is upregulated during heat and oxidative stress. When *jwa* is underexpressed, flies exhibit defects in tolerance, whereas they show increased tolerance when *jwa* is overexpressed [57]. The prevalence of stress-response genes affecting alcohol-related behaviors supports the notion that stress is related to addiction behaviors across species (Figure 2).

Unsurprisingly, genes associated with the regulation of gene expression are also associated with tolerance phenotypes. These connections suggest the possibility of tolerance mechanisms arising from epigenetic alterations. Genes implicated in transcription, such as *nejire*, which encodes for a histone acetyltransferase, were also shown to modulate ethanol tolerance. Flies with *nejire* knockdown have reduced tolerance, whereas *nejire* overexpression mimics tolerance [58]. Similarly, the transcriptional regulator *NO66* encodes a histone demethylase with a domain for *JmjC* (Jumonji), and *NO66* knockdown flies demonstrate enhanced tolerance on a dose-dependent basis [59]. Regulators of translation such as *rluA-1*, *pumilio*, and *krasavietz* are also known to modulate tolerance [60]. *rluA-1* encodes an enzyme, which catalyzes RNA nucleoside modification [61]; *pumilio* encodes an RNA-binding protein and functions as an RNA decay-mediated translational repressor [62]; and *krasavietz* encodes a protein that interacts with a translation initiation factor [63]. Mutants of all three lead to reduced ethanol tolerance [17]. These particular genes suggest that RNA modulation at the synapse plays a role in tolerance (Figure 1).

There has been an emphasis on the role of neurotransmission and synaptic activity in modulating EIBs, with a particular focus on tolerance. Several genes involved in synaptic vesicle formation and exocytosis were also identified as important to ethanol tolerance. Shibire, the *Drosophila* homolog of mammalian Dynamin [64], is involved in endocytosis and vesicle recycling; Syntaxin 1A, which is involved in exocytosis [65]; and Synapsin, which controls a phosphoprotein in the vesicle [66], have all been shown to modify ethanol tolerance. Both *shibire* and *syntaxin 1A* mutants reduce ethanol tolerance, while *synapsin* mutants show enhanced tolerance [67,68]. While the mechanism of modulation is not known, there appears to be a clear association between the synaptic vesicles and ethanol tolerance.

Genes associated with neurotransmitter function have emerged as an important category of tolerance regulators. Homer, which interacts with group 1 glutamate receptors, modifies sleep and locomotion and decreases ethanol tolerance when mutated [69]. Octopamine, which is a neurotransmitter/neurohormone, is encoded by *tyramine beta hydroxylase* (*tβh*) and appears to be required for ethanol tolerance. This is evident in *tβh* mutant flies, which show reduced tolerance [18]. Rogdi, an atypical leucine zipper, is involved in GABA transmission and was shown to reduce tolerance when mutated [60] (Figure 2).

## 5. Circadian Rhythm Genes Associated with Ethanol Tolerance

Several studies in *Drosophila* have identified an association between some genes involved in circadian rhythm and modulation of EIBs [70,71,72,73,74]. The circadian genes were originally of particular interest because ethanol sensitivity is known to fluctuate during different phases of the sleep–wake cycle for both humans and *Drosophila*, implicating their connection in ethanol processing [75]. Circadian genes remain salient because their expression levels alter in a 24 h cycle, and the epigenetic response to stimuli is highly significant in ethanol research, particularly when it comes to tolerance [73]. These factors have made circadian rhythm genes an important focus in the field of rapid tolerance development, and while not much is known about their mechanisms, their role in tolerance modulation is apparent. It is important to note the extent to which circadian-related genes modify tolerance, with most genes causing complete abolition of the tolerance phenotype when knocked out (Table 1). The goal of this review is to highlight the apparent relationship between genes that modulate circadian rhythm and tolerance to ethanol.

Several studies have explored the effects of circadian rhythm genes on tolerance in *Drosophila*, with particular focus on the four core circadian genes: *period* (*per*), *timeless* (*tim*), *cycle* (*cyc*), and *clock* (*clk*). Mutated forms of all four core circadian rhythm genes—*per^01^*, *tim^01^*, *cyc^01^*, and *clk^JRK^*—disrupt the circadian 24 h cycle [76], and all have been studied extensively for their effect on EIBs [70,72,73,74]. Mutant alleles of circadian genes *per^01^*, *cyc^01^*, and *tim^01^* fail to develop rapid tolerance to ethanol [73]. In addition, disruption of *per* and *tim* expression via exposure of flies to constant light also resulted in failure to develop rapid tolerance [73]. However, tolerance is not affected in *clk^JRK^* mutant flies. Furthermore, it was suggested that tolerance acquisition is not linked to the phases of the circadian cycle in both wild type and circadian gene mutant flies [74]. Taken together, these findings indicate that the pathway by which these circadian rhythm gene mutations affect ethanol tolerance might be separate from the pathway by which their mutations affect circadian rhythms [73,74]. Nevertheless, the unresolved connections between circadian rhythm genes and tolerance modification merits further investigation. Beyond these core circadian rhythm genes, several other genes have been implicated in both circadian rhythm and ethanol EIB modulation, which strengthens the possibility of a relationship between these two complex processes.

*slowpoke* (*slo*) encodes BK channels, which are found in many cell types and are a known target of ethanol. Flies with nonfunctional *slowpoke* in neuronal tissues experience circadian arrhythmicity [77,78]. Flies with the *slo* null allele, *slo^4^*, are unable to develop tolerance to ethanol, suggesting that BK channels are required for ethanol tolerance. BK channels were also shown to influence the response to other volatile inhalants, indicating that the mechanism through which ethanol interacts with BK channels is broadly utilized [77]. Another study observed that exposure to benzyl alcohol causes the expression of slowpoke, suggesting that benzyl alcohol-induced overexpression of BK channels is associated with the generation of rapid tolerance [79].

*dCreb2* encodes Cyclic AMP Response Element-Binding protein, a transcription factor that regulates the expression of BK channels. A loss-of-function allele of *dCreb2* inhibits the tolerance phenotype caused by BK channels because CREB2 upregulates *slo* expression following drug exposure. Although this study used benzyl alcohol, it is likely that the mechanism for ethanol regulation will be similar [80]. Modification of tolerance by *nejire* is also likely associated with this pathway, as it is known to be recruited by CREB2. Moreover, a ChIPseq-based study has shown the interaction of Nejire with BK channels and Pumilio [58].

*Hr38* encodes the *Drosophila* ortholog of human immediate early response transcription factors Nr4a1/2/3. It is hypothesized that Hr38 modulates circadian rhythm by acting as a link between *clk* expression and the transcription of other genes involved in circadian modulation [81]. Overexpression of Hr38 increases ethanol tolerance, whereas knockdown mutants show decreased tolerance. Expression of *Hr38* is induced following acute exposure to ethanol, but levels return to baseline within three hours, suggesting that the upregulated presence of this protein alone cannot explain the tolerance phenotype [82].

*discs large* (*dlg1*) encode a membrane-associated guanylate kinase, which is a homolog of mammalian PSD-95 and SAP97, and affects both EIBs and circadian rhythm when mutated [83]. While the function of *dlg1* in the adult fly CNS is unclear, it was implicated in the control of circadian sleep cycles because null mutations of this gene caused changes in daytime sleep patterns [84,85]. Nevertheless, *intolerant*, a null allele of *dlgS97*—a neuronal isoform of *dlg1*, which is an essential part of the protein complex for NMDA receptor subunit dNR1, does not develop normal levels of tolerance. This receptor plays an important role in synaptic plasticity, suggesting that Dlg1 affects ethanol tolerance via synaptic plasticity [83].

**Table 1:** Association of circadian-related genes with ethanol tolerance.

Gene	Reduction in Tolerance (%)	Function	Role in Circadian Rhythm	Other Phenotypes
***per***	100 [73]	Forms dimer with TIM to inhibit function of CLK/CYC	Core protein	
***tim***	100 [73]	Forms dimer with PER to inhibit function of CLK/CYC	Core protein	
***cyc***	100 [73]	Transcription factor for *per* and *tim*	Core protein	
***slo***	100 [77]	BK channel	Downstream of core proteins	Anesthetic sensitivity
***dCREB2***	100 [80]	Transcription factor that regulates the expression of BK channels	Upregulation of *slo* expression	Learning, memory, and sexual behavior
***Hr38***	50 (approx.) [82]	Immediate early response transcription factor	Transcription factor downstream of CLK	Ethanol preference
***dlg1***	30 (approx.) [83,85]	Membrane-associated guanylate kinase	Regulation of morning peak, sleep	Courtship

## 6. Future Perspectives

Over the last three decades, the evidence supporting the role of genetic factors on ethanol tolerance has expanded considerably, with more than 90 studies on the topic published since 1997. The genes in these studies represent many different functions in the CNS. It is interesting that genes associated with certain processes—such as stress response, regulation of gene expression, neurotransmission, and particularly, circadian rhythm—have differentially appeared as modulators of tolerance behavior. Most of the current research provides descriptions of the genetic function, and some studies have indicated a connection between this function and modulation of tolerance, but very few studies are able to explain the mechanism of modulation for these genes. One of the goals of this field of research is to alleviate the notable deficiency in our understanding of the neuronal basis of tolerance behavior and its genetic underpinning. To identify the mechanisms, many recent studies have understandably explored the manifestation of tolerance as a result of rapid modulation of gene expression. An important aspect of this research is to understand the timeline of modification of the tolerance phenotype. While many studies choose 24 h as the time point at which to measure tolerance change, understanding the time it takes for this tolerance change to develop and disappear—if it does disappear at all—is crucial to our understanding of the mechanisms by which tolerance acts.

The genes discussed in this review are likely the tip of the genetic iceberg and we expect further studies will identify additional genes and pathways associated with tolerance modulation. The current research lays important groundwork for understanding the mechanisms by which tolerance is altered by genetics, and further exploration of this process will help us to understand not only the underlying structures of AUDs but also more broadly, the processes of addiction, learning, and memory.

## 7. Conclusions

After decades of research into the genes that alter ethanol tolerance in *Drosophila*, it is clear that genes associated with stress pathways, regulation of gene expression, neurotransmission, and circadian rhythm play a significant role in establishing ethanol tolerance (Figure 2). The influence of genes associated with diverse functions on ethanol tolerance suggests the involvement of various mechanisms in the development of tolerance. Although core- and circadian-associated genes have emerged as a group strongly associated with tolerance, the mechanisms underlying this relationship remain largely unknown (Table 1). Knockdown of these genes generally results in a reduction in tolerance. It will be important to assess whether these phenotypes are due to initial resistance to sedation and/or the development of tolerance after initial exposure to ethanol [54]. The aim of this review is to emphasize the need for further research into the relationship between circadian rhythm and ethanol tolerance. There is no doubt that the pathways of tolerance modulation are complex and multifaceted, but the current research provides a solid foundation for understanding these mechanisms.
